# The need to address the myopia pandemic: summary report of the global myopia public health summit 2024

**DOI:** 10.1186/s41256-025-00445-7

**Published:** 2025-09-23

**Authors:** Wei Pan, Ian Morgan, Ian Flitcroft, Kathryn Rose, Lisa A. Ostrin, Mark Rosenfield, Pirindhavellie Govender-Poonsamy, Dunia Siu-Villaseñor, Hakan Kaymak, Jong Mei Khew, Oliver Woo, Kazuo Tsubota, Lakshmi Shinde, Olga Prenat, Jun Ma, Jia Qu, Zhikuan Yang, Yan Wang, Kah Ooi Tan, Amanda Davis, Weizhong Lan

**Affiliations:** 1 Aier Academy of Ophthalmology, Central South University, Changsha, China; 2 Research School of Biology, College of Medicine, Biology and Environment, Australian National University, Canberra, Australia; 3 State Key Laboratory of Ophthalmology, Guangdong Provincial Key Laboratory of Ophthalmology and Vision Science, Guangdong Provincial Clinical Research Center for Ocular Diseases, Zhongshan Ophthalmic Centre, Sun Yat-Sen University, Guangzhou, China; 4 Ophthalmology Department, Children’s Health Ireland at Temple Street Hospital, Dublin, Ireland; 5 Centre for Eye Research Ireland, Technological University of Dublin, Dublin, Ireland; 6 Graduate School of Health, University of Technology Sydney, Sydney, Australia; 7 University of Houston College of Optometry, Houston, TX USA; 8 SUNY College of Optometry, New York, NY USA; 9 Department of Optometry, University of KwaZulu Natal, Durban, South Africa; 10 Autonomous University of Sinaloa, 80010 Culiacan, Sinaloa Mexico; 11 University Eye Hospital Homburg/Saar, Homburg, Germany; 12 Breyer Kaymak Klabe Augenchirurgie, Düsseldorf, Germany; 13 Asia Optometric Congress, Singapore, Singapore; 14 Asia Optometric Management Academy, Hong Kong, China; 15 Tsubota Laboratory, Inc., Tokyo, Japan; 16 Keio University School of Medicine, Tokyo, Japan; 17 Shinde Eye Care, IACLE, Bangalore, India; 18 Essilorluxottica Medical Affairs, Charenton-le-Pont, France; 19 School of Public Health, Institute of Child and Adolescent Health, Peking University, Beijing, China; 20 National Engineering Research Center of Ophthalmology and Optometry, National Clinical Research Center for Ocular Diseases, Eye Hospital, Wenzhou Medical University, Wenzhou, China; 21 Oujiang Laboratory, Zhejiang Laboratory for Regenerative Medicine, Vision and Brain Health, Wenzhou, Zhejiang China; 22 Changsha Aier Eye Hospital, Aier Eye Hospital Group, AIER Mansion, No.188 South Furong Road, Changsha, 410000 Hunan Province China; 23 Department of Child and Adolescent Health Promotion, Jiangsu Provincial Center for Disease Control and Prevention, Nanjing, China; 24 International Association of Contact Lens Educators (IACLE), Asia-Pacific, Bangalore, India; 25 International Agency for Prevention of Blindness, London, UK; 26 The Fred Hollows Foundation, Sydney, Australia

**Keywords:** Review, Global, Public health, Myopia, Management, Strategies

## Abstract

The World Health Organization (WHO) recognizes myopia as a significant public health concern, as its prevalence has been rising at an alarming rate worldwide. In the WHO’s Global Action Plan for the Prevention of Avoidable Blindness and Visual Impairment 2014–2019, myopia has already been identified as a major target for action. However, the attention paid and measures taken to treat this target is significantly diverse across the globe. This report presents the summary of the Global Myopia Public Health Summit jointly organized by the International Agency for the Prevention of Blindness (IAPB), the Asia Optometric Congress (AOC), and Aier Eye Hospital Group in September 2024. The summit centered on the public health, clinical challenges, and practical barriers faced in different regions of the world, along with strategic recommendations for myopia prevention and control. The summit has concluded: (1) although myopia prevalence is rising globally, population-based data remain limited in many regions; (2) common challenges such as limited awareness of myopia, the high cost of interventions, and the lack of continuing education for practitioners should be addressed; (3) while effective interventions are crucial for controlling myopia, their cost-effectiveness needs to be evaluated both at the individual and societal levels; (4) countries like China, where government-led initiatives have integrated school vision screening, reduction of education burden, compulsory increase of outdoor activities, and advocacy for eye health awareness, offer valuable experience and lessons for other countries or regions facing similar myopia epidemics.

## Introduction

Myopia has become a significant public health issue in many regions worldwide. In the World Health Organization’s Global Action Plan for the Prevention of Avoidable Blindness and Visual Impairment 2014–2019, myopia has already been identified as a major target for action [1]. It has been projected that by 2050, 50% of the global population will be myopic (Spherical Equivalent: SE ≤ −0.50D) and 10% will be highly myopic (SE ≤ −5.00D) [2]. However, detailed and accurate prevalence estimates are still lacking for many countries and regions. Myopia, particularly high myopia, increases the risk of developing severe ocular conditions such as retinal detachment, myopic macular degeneration (MMD), and other pathologies that cannot be optically corrected, potentially leading to severe visual impairment and blindness [3]. In 2015, the global productivity loss due to visual impairment from uncorrected myopia was estimated at $244 billion USD, which is about 0.3% of world GDP, with $6 billion USD attributed to MMD [4]. Effective interventions for myopia prevention and control are crucial. Given that myopia prevalence can range from 40 to 90% in certain regions, cost-effectiveness must be considered not only at the individual level but also at the population level.

**Fig. 1:**
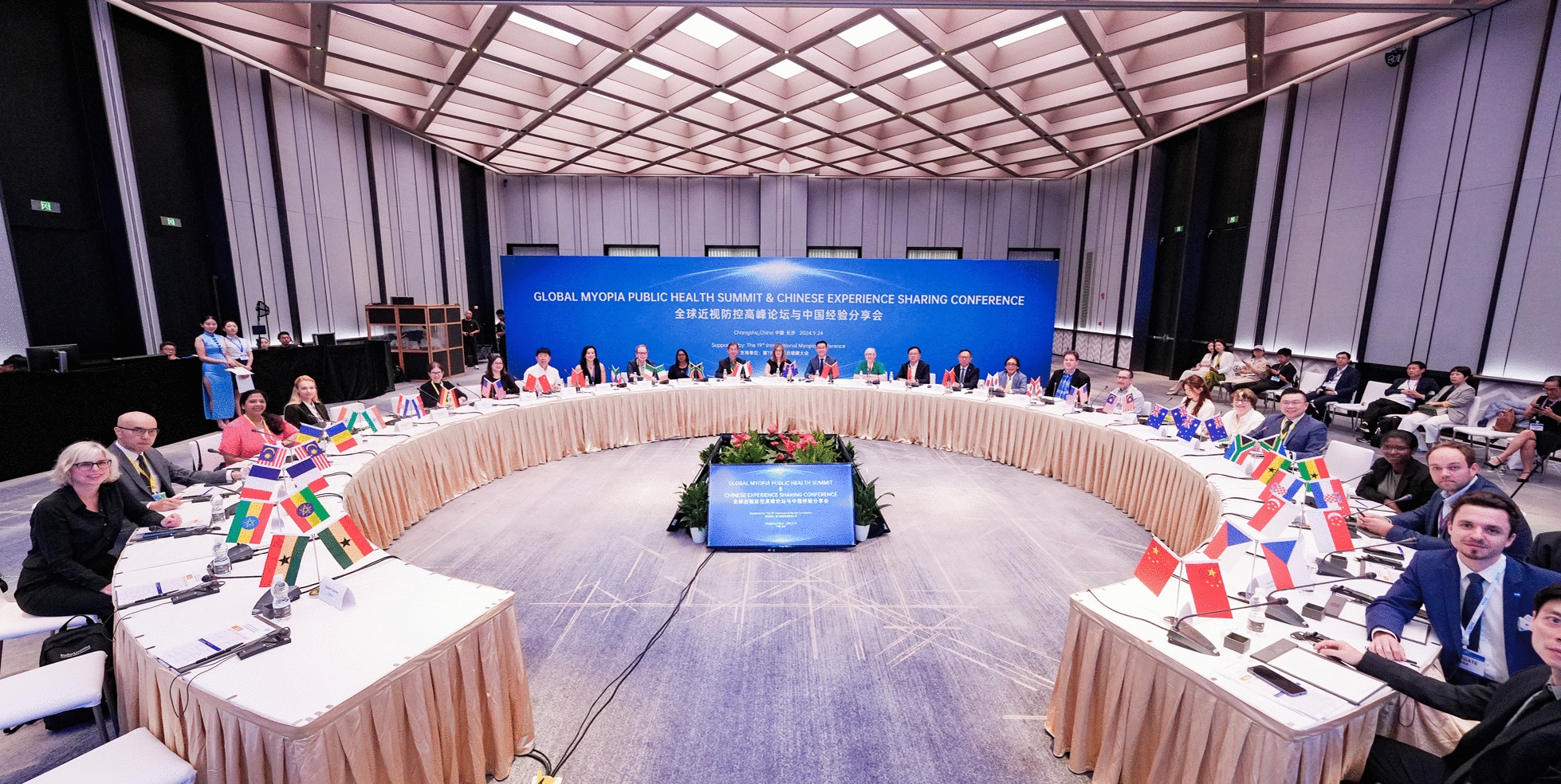
Group photo of the Global Myopia Public Health Summit & Chinese Experience Sharing Conference. (Copy right © 19th International Myopia Conference)

In September 2024, the International Agency of Prevention of Blindness (IAPB) and the Asia Optometric Congress (AOC) organized the ‘Global Myopia Public Health Summit’, during the Pre-meeting of 19th International Myopia Conference hosted by Aier Eye Hospital Group in Changsha, China [5]. The summit gathered 31 experts from ophthalmology, optometry, health economics, Non-Governmental organizations (NGOs), industry, and government from 17 countries across North America, Europe, Oceania, Asia, South America, and Africa to participate (Fig. 1). The purpose of this summit was to: (1) share and discuss public health and practical challenges in myopia management across various countries and regions; (2) explore accountable, affordable, and accessible strategies for managing myopia globally; and (3) share China’s experience and efforts in combating myopia. In this article we aim to summarize the key discussions and outcomes of the summit.

## Global myopia prevalence

Myopia prevalence has been estimated in various populations across the globe. When discussing myopia prevalence, it is essential to analyse whether the data are derived from refractive error measurements with or without cycloplegia, especially in children. Cycloplegic data should not be directly compared with non-cycloplegic data, as non-cycloplegic measurements tend to overestimate myopia prevalence in children. For example, the Shandong Eye Study, which included 11,990 children aged 4 to 18 years, found a 17.3% difference in myopia prevalence between cycloplegic and non-cycloplegic assessments (52.2% without cycloplegia vs. 34.9% with cycloplegia) [6]. Nevertheless, both cycloplegic and non-cycloplegic findings underscore the rising public health concern of myopia globally. In many countries and regions, it is more practical to screen for myopia using non-cycloplegic refractive error measurements or visual acuity tests. In this review, unless otherwise specified, prevalence data were based on non-cycloplegic measurements.

### Myopia prevalence in East Asia, Singapore, and association with education

The prevalence of myopia and high myopia in China, Japan, South Korea, and Singapore is significantly higher than in other parts of the world. The estimated prevalence of myopia and high myopia is around 80–90% and 10–20% in 17–18 year old [7]. This rise began after the Second World War, when the countries of the region started to rapidly develop economically, based in part of the development of a highly educated labour force. The rapid rate of increase was too high to be attributed to genetic changes [7]. Education burden, particularly from an early age, and limited time spent outdoors are the two main contributing factors [8]. Regions with modern East Asian education systems showed much higher myopia prevalence than the regions with modern Western education, limited formal education, or no formal education [7]. Positive associations between myopia prevalence and the Program in Student Assessment (PISA) scores were also found [9].

### Myopia prevalence in North America, South America, and Africa

Contemporary population-level myopia prevalence data in North and South America are limited, the most recent data from United States covers the period from 1999–2004 [10, 11]. The prevalence of myopia in the United States (age from 18 to 24 year old) rose significantly, from 27.7% in 1971–1972 to 38.1% in 1999–2004, with high myopia experiencing an eight-fold increase over the same timeframe [11]. More recent and accurate data is needed, as the method used define myopia in 1971–1972 was mainly based on visual acuity, and the autorefraction measurement in 1999–2004 was non-cycloplegic [11]. In South America, a meta-analysis conducted by Guedes et al. found that the overall prevalence of myopia among South American children and adolescents (3 to 20 year old) was 8.6%. However, the number of studies included in the analysis was relatively low (N = 38), and the reported prevalence across these studies ranged widely from 0.80% to 47.36% [12]. In Africa, a meta-analysis by Kobia-Acquah et al. reported a myopia prevalence of 4.7% and a high myopia prevalence of 0.6% among children (age from 5 to 18 year old) [13]. These findings align with another meta-analysis by Ovenseri-Ogbomo et al. published in the same year [14]. Regionally, myopia prevalence in Africa was 6.8% in Northern Africa, 6.3% in Southern Africa, 4.7% in Eastern Africa, and 3.5% in Western Africa [13].

## Barriers and challenges facing myopia prevention and control

Population-level data on myopia prevalence remains scarce across many regions and countries, yet an increasing myopia problem is clear. Common themes of barriers and challenges facing myopia prevention and control in each regions includes: (1) limited awareness and attitudes towards myopia among patients, the general public, and policymakers; (2) affordability and accessibility of myopia management interventions; and (3) a lack of continuing education for practitioners.

### Lack of awareness

Low awareness of myopia is a global issue. In Ghana, only 49.2% of 1919 surveyed adults had heard of myopia [15]. In Israel, a study of 161 Ultra-Orthodox Jewish parents found that while only 7% viewed myopia as a disease, 89% believed that its progression should be treated, yet just 22% were aware of available treatment options [16]. In Saudi Arabia, among 358 surveyed parents, 38.3% had never heard of refractive error, 33.8% did not believe it could lead to visual impairment, and 63.7% reported never receiving any information about myopia during pediatric clinic visits [17]. In Spain, among 321 surveyed parents, nearly 40% of them didn’t know any myopia control strategies, and the main source of information is from relatives and practitioners [18]. In Germany, a survey of 44 patients undergoing SMILE (Small Incision Lenticule Extraction) refractive surgery revealed that 67.4% were unaware of their refractive status before the procedure [19]. According to the International Myopia Institute (IMI) 2022 clinical practice survey, single-vision spectacles remain the most commonly prescribed intervention for myopia correction worldwide, despite their lack of efficacy in slowing progression [20].

### Affordability and accessibility

Challenges related to cost and access are not confined to low-income countries. In the United States, for example, spectacle-based interventions such as highly aspherical lenslets (HAL) and defocus incorporated multiple segments (DIMS), as well as low-dose atropine, have not yet received FDA approval for myopia control. Most health insurance plans do not cover these treatments, making them financially burdensome for many families.

### Lack of continuing professional education

Many practitioners globally remain under-informed about evidence-based strategies for myopia control. In an international survey of 3,195 practitioners, a significant proportion still considered outdated methods effective: 58.7% in Africa, 31% in Asia, 13.2% in Europe, 18.4% in North America, and 37.3% in South America believed that under-correction or single-vision spectacles were valid myopia control approaches, [20] despite evidence showing these methods are ineffective in slowing progression [21, 22].

In the coming years, the standard of care is expected to shift, placing greater emphasis on myopia management [23]. This will likely require more frequent and longer clinical visits [23, 24]. An interesting study assessing the workforce implications of implementing myopia management strategies, as opposed to traditional one-time refractive error correctio, highlighted an increased burden on eye care professionals in the UK and Ireland, particularly in the short term [23]. However, the study concluded that with targeted workforce expansion and additional training for current practitioners, eye care services would be able to meet the increased demand without becoming overwhelmed [23].

## Myopia management and strategies

### Clinical efficacy and cost-effectiveness strategies in myopia management

Given the wide range of anatomical changes associated with myopia (e.g., axial elongation, choroidal thinning), there is no “safe” level of myopia. Even low levels of myopia are linked to an increased risk of sight-threatening ocular diseases [3, 25, 26]. For example, findings from the Australian Blue Mountains Eye Study revealed that 43% of individuals with myopic maculopathy had myopia of less than − 5.00 diopters [27, 28]. Importantly, modeling studies suggest that slowing myopia progression by just 1 diopter could reduce the risk of developing myopic maculopathy by approximately 40% [28]. Growing evidence suggests that multiple modalities are effective in controlling myopia progression. A meta-analysis by Lawrenson et al. compared the clinical efficacy of various myopia control options, demonstrating that multifocal spectacles, multifocal soſt contact lenses, low-dose atropine (concentration of 0.01% to 0.05%), peripheral plus spectacles, and Orthokeratology (ortho-k) lenses all showed promising results [29]. In one year of treatment, axial length elongation compare to single-vision spectacles, multifocal spectacles was − 0.06 (95% CI − 0.09, − 0.04) mm less, multifocal soſt contact lenses was − 0.11 (95% CI − 0.13, − 0.09) mm less, low-dose atropine 0.05% was − 0.13 (95% CI − 0.21, − 0.05) mm less, peripheral plus spectacles was − 0.13 (95% CI − 0.24, − 0.03) mm less, and ortho-k was − 0.19 (95% CI − 0.23, − 0.15) mm less [29]. When considering the lifetime costs associated with severe myopia-related complications (e.g., retinal detachment, myopic macular degeneration), studies by So et al. [30] and Fricke et al. [31] found that active treatments such as HAL, DIMS, low-dose atropine, and ortho-k were more cost-effective than single-vision spectacle treatment. Importantly, increasing outdoor time, a simple, no-cost, and evidence-based strategy, remains one of the most effective population-level measures for myopia prevention and should be widely promoted.

### China’s experience

In 2012, Li et al. estimated that, without the rapid development of refractive services in China, uncorrected myopia could cost the country 1–3% of its GDP due to lost productivity [32]. Since 2018, China has prioritized myopia prevention and control at the national level by implementing a series of comprehensive policies and strategies. First, reducing the prevalence of myopia was established as a key performance indicator (KPI) for local governments, encouraging coordinated action among government departments, schools, medical institutions, media, families, and students [33]. Second, nationwide myopia screening is now conducted twice a year, [34]. enabling parents to access updated information on their children’s refractive status. In most schools, health education sessions related to myopia prevention are organized at least once per semester for both parents and students to raise awareness and promote proactive measures. Third, to improve the affordability of effective interventions, the government has pursued volume-based procurement strategies to reduce the cost of ortho-k lenses [35]. Fourth, the “Double Reduction” policy was introduced to alleviate students’ academic pressure by reducing homework and restricting after-school tutoring. This policy aims to lessen students’ academic burden and potentially increase their outdoor activities [36]. However, despite these efforts, empirical evidence on the long-term effectiveness of these national strategies in reducing myopia prevalence remains limited. More time and further evaluation are needed to assess their true impact.

China’s Taiwan region began implementing large-scale myopia prevention and control efforts earlier than China’s Mainland [37]. In the 1980s, the China’s Taiwan region launched the Taiwan Student Vision Care Program (TSVCP) [37]. The program began with routine school-based vision screenings every semester. Over the years, multiple epidemiological researches, treatment programs, and environmental interventions, such as improving classroom lighting and adjusting desk and chair heights were tested and implemented [37]. However, these early efforts had limited success, likely due to the lack of evidence-based strategies at the time. In 2010, the “Tian-Tian 120” initiative was introduced, advocating for at least 120 min of outdoor activity daily for schoolchildren [37–39]. Following its implementation, China’s Taiwan region observed a meaningful decline in myopia prevalence, from 50% in 2010 to 46.1% in 2015 among primary school students [39].

China’s Mainland and China’s Taiwan region share several common approaches in combating myopia. First, epidemiological evidences, such as high myopia prevalence, rapid growth trends, and significant economic burden, drew the attention of both education and health policy makers. Second, both regions implemented some form of school-based vision screening, including non-cycloplegic autorefraction or visual acuity testing. Third, they promoted large-scale, low-cost but effective interventions, particularly by encouraging increased time spent outdoors.

For countries or regions lacking robust epidemiological data but concerned about rising myopia prevalence, a critical first step is to recognize vision health as a fundamental economic, social, and developmental issue. This perspective can catalyze government engagement in myopia prevention and control, ultimately contributing to the reduction of avoidable vision loss. Additionally, the World Health Organization has launched a free mobile application called WHOeyes, which serves as a practical tool for myopia screening, particularly in resource-limited settings.

## Conclusions

The global rise in myopia presents a significant public health challenge with far-reaching social and economic consequences. Representatives from regions lacking research data on myopia prevalence voiced strong concern about the increasing trend of myopia and the variable levels of public and governmental attention. While numerous myopia management interventions have proven effective, their accessibility and affordability remain challenging in various countries and regions. The Chinese experience underscores the potential of government-led initiatives to reduce myopia prevalence through comprehensive policies, mass screenings, and educational campaigns. There is strong hope for increased engagement from NGOs, the World Health Organization (WHO), industry stakeholders, and professional societies to address these gaps, raise awareness, and advocate for action. Moving forward, addressing the gaps in data and cost estimates, along with enhancing the capacity of practitioners and raising public awareness, will be crucial to controlling the impact of myopia pandemic. Building the evidence, taking collective action and a uniformity of messaging and approach to high level political and health forums and governments is critical to ensure policy and action across promotive, preventative, treatment and rehabilitation for myopia and high myopia is prioritized and financed.

## Data Availability

Not applicable.
